# *Cannabis Sativa* targets mediobasal hypothalamic neurons to stimulate appetite

**DOI:** 10.1038/s41598-023-50112-5

**Published:** 2023-12-27

**Authors:** Emma C. Wheeler, Pique Choi, Joanne De Howitt, Sumeen Gill, Shane Watson, Sue Yu, Peyton Wahl, Cecilia Diaz, Claudia Mohr, Amy Zinski, Zhihua Jiang, David Rossi, Jon F. Davis

**Affiliations:** 1 Department of Integrative Physiology and Neuroscience, Washington State University, Room 115, Veterinary Biomedical Research Building, Pullman, WA 99164 USA; 2 Department of Animal Sciences, Washington State University, Pullman, WA USA

**Keywords:** Neuroscience, Feeding behaviour, Motivation, Feeding behaviour

## Abstract

The neurobiological mechanisms that regulate the appetite-stimulatory properties of *cannabis sativa* are unresolved. This work examined the hypothesis that cannabinoid-1 receptor (CB1R) expressing neurons in the mediobasal hypothalamus (MBH) regulate increased appetite following cannabis vapor inhalation. Here we utilized a paradigm where vaporized cannabis plant matter was administered passively to rodents. Initial studies in rats characterized meal patterns and operant responding for palatable food following exposure to air or vapor cannabis. Studies conducted in mice used a combination of in vivo optical imaging, electrophysiology and chemogenetic manipulations to determine the importance of MBH neurons for cannabis-induced feeding behavior. Our data indicate that cannabis vapor increased meal frequency and food seeking behavior without altering locomotor activity. Importantly, we observed augmented MBH activity within distinct neuronal populations when mice anticipated or consumed food. Mechanistic experiments demonstrated that pharmacological activation of CB1R attenuated inhibitory synaptic tone onto hunger promoting Agouti Related Peptide (AgRP) neurons within the MBH. Lastly, chemogenetic inhibition of AgRP neurons attenuated the appetite promoting effects of cannabis vapor. Based on these results, we conclude that MBH neurons contribute to the appetite stimulatory properties of inhaled cannabis.

## Introduction

It is well established that *cannabis sativa* promotes appetite^1–4^, and this realization has guided the development of synthetic compounds to treat anorexia in a range of clinical applications^5,6^. However, therapies that mimic the pharmacological action of individual phytocannabinoids are not well tolerated and often fail to reliably stimulate feeding^7–9^. As a result, a disproportionate number of patients with anorexia use *cannabis sativa* to promote appetite^10–14^, although remarkably, the neurobiological mechanisms supporting this process remain largely unknown.

Prior work has established that neurons within the mediobasal hypothalamus (MBH), namely the arcuate nucleus (ARC), regulate diverse aspects of feeding behavior^15–17^. Previous work from our lab indicates that exposure to cannabis vapor stimulates appetite, hedonic consumption of palatable food and mRNA expression changes in the MBH of rodents^18^. Agouti related peptide (AgRP) neurons in the MBH are necessary and sufficient to stimulate food intake, making them a logical target of inhaled *cannabis sativa*^19^. Notably, AgRP afferent terminals express the cannabinoid type-1 receptor (CB1R), a prominent regulator of cellular signaling by endogenous cannabinoids and phytocannabinoids^20^. Collectively, these observations support the hypothesis that CB1Rs within MBH neurons contribute to the appetite-stimulatory properties of inhaled *cannabis sativa*. To test this hypothesis we measured meal patterns, locomotor activity, operant responding, in vivo calcium dynamics of MBH neurons, ex-vivo patch-clamp electrophysiological recordings and conducted chemogenetic manipulations of AgRP neurons in rodents exposed to cannabis vapor.

## Materials and methods

### Animals

Male Long-Evans rats (Envigo, IN) weighing 275-300 g, male C57BL/6J mice weighing 20-30 g (Jackson Laboratory, ME), *Agrp*^*tm1(cre)Low1*^*/J* (C57BL/6J background) mice weighing 20-30 g were used as experimental subjects. Rats were initially used as the model species due to the ease of behavioral assays, however we also made use of multiple mouse models which allowed for mechanistic imaging and chemogenetic studies. All rodents were maintained on ad libitum food and water except when indicated. Rodents were housed in an environmentally controlled vivarium on a reverse 12:12 light cycle. All animal groups were randomly selected via a random number generator for equal mean body weights for each experiment in this study. One rat was excluded from the study due to large outlying data points in all measurements taken. All work adhered to the guidelines and regulations and was approved by the Institutional Animal Care and Use Committee (IACUC) guidelines at Washington State University. Additionally, experiments in this study are reported in accordance with ARRIVE guidelines.

### Cannabis plant

In accordance with federal law, the *Cannabis sativa* plant matter used in this study was obtained under federal guidelines with a Drug Enforcement Agency Schedule I drug license from the National Institute on Drug Abuse (NIDA) via Research Triangle. The ground whole plant *Cannabis sativa* used contained 7.8% THC and 0.5% CBD.

### General procedure

#### Rat studies

For each experiment, rats (*n* = 8/group) were habituated to the vapor chambers for 10 min across 5 days prior to any experimental manipulation. On test days, rats were exposed to cannabis plant matter (7.8% THC, 0.5% CBD, NIDA, Research Triangle, NC) vaporized over a 10-min period. Control rats were placed into an identical apparatus with an unfilled vaporizer and served as “air-treated” controls. We used a behaviorally relevant cannabis dose (800 mg) known to stimulate food intake^18^. A second cohort of free-feeding rats (*n* = 8/group) was habituated to BioDAQ metabolic chambers for 10 days prior to any experimental manipulation. After habituation, rats were exposed to 800 mg of cannabis and feeding patterns were measured in the BioDAQ metabolic chambers over a 4-h period. In addition, open-field exploration was used to assess acute locomotor activity in a subset of rats (*n* = 4) 30 min after cannabis exposure in this experiment. A third cohort of free-feeding rats (*n* = 10) was habituated to Sable Promethion metabolic chambers for 10 days. After habituation, feeding patterns and locomotor activity were measured in the Sable Promethion metabolic chambers over a 4-h period following cannabis exposure. A fourth cohort of rats (*n* = 6/group) was tested for food-reinforced behavior following cannabis exposure using bussey touch screen chambers.

### Experiment 1: cannabis-induced feeding pattern analysis in free feeding rats

To measure the effects of cannabis on meal patterns, one cohort of rats were habituated to BioDAQ chambers (*n* = 8/group) and a second cohort of rats were habituated to the Sable Promethion metabolic (*n* = 6 experimental and *n* = 4 control) chambers for 10 days prior to any experimental manipulation. During this process, rats were introduced to the vapor chambers without any cannabis exposure to eliminate the effects of stress due to handling. On test days, rats received an 800 mg dose of vaporized cannabis plant matter and were immediately returned to the BioDAQ chambers or Sable system chamber. Subsequently, feeding measurements were taken continuously for 4 h to examine temporal changes in meal patterns. Meals were defined as 0.2 g or larger separated by 10 min.

### Experiment 2: cannabis effects on locomotor activity

An open-field arena was used to quantify locomotor activity following cannabis exposure. The field measured 45 cm by 45 cm and was constructed with a white floor and transparent plexiglass walls around the perimeter, leaving the top of the field exposed to the room. A camera was stationed directly above the field to capture locomotor activity. Rats were habituated to the field environment for 10 min on three separate days. Subsequently, rats received 800 mg of vaporized cannabis and were placed in the open-field arena 30 min later (*n* = 4/group). Locomotor activity was evaluated by measuring total movement and total distance traveled over the 30 min period with Ethovision XT 11.5 software. Separately, the locomotor activity of a second cohort of rats living in Sable Promethion metabolic chambers (*n* = 6 experimental and *n* = 4 control) was monitored following 800 mg of vaporized cannabis. Locomotor activity was evaluated by measuring total movement and total distance traveled over a period of 24 h and later segmented to the second 30 min following vapor cannabis exposure. Metabolic measurements of Energy Expenditure (EE), Respiratory Exchange Rates (RER), rate of oxygen consumption (VO_2_), and rate of carbon dioxide emission (VCO_2_) were also gathered from rats in the Sable Promethion chambers. RER was calculated using the Weir Equation [Energy Expenditure (kcal/hr) = 3.941(VO_2_) + 1.106(VCO_2_)]^21^. Measurements were grouped by rat and combined into three minute bins for the 30 min to 1 h post-exposure timeframe for analysis.

### Experiment 3: operant responding following cannabis exposure

The effects of cannabis on food-reinforced behavior were examined using a rodent touch screen operant system. In this experiment, rats were trained to respond for 45 mg sucrose pellets in a Bussey Touch Screen System (89547-R-Lafayette Instrument Company, Lafayette, IN), as described previously^18^. Rats maintained on ad libitum chow were trained using this protocol until they reached a sufficient response (15 correct touches) over a 30-min period (5 days). Following acquisition of food-reinforced behavior, one cohort (*n* = 6/group) of rats was placed into the chambers 1 h after air or cannabis vapor exposure (800 mg). To determine if the effects on food-seeking were time-dependent, on a second day, rats was placed into the chambers 2 h after air or cannabis exposure. During each experiment, food was removed from the home cages during drug assimilation (1 h or 2-h period) and subsequently, correct touches, blank touches, correct touch latency, and blank touch latency were recorded over a 30-min period for each condition.

### Mouse Studies

#### Experiment 4: dose response to cannabis in mice.

In this experiment we established a behaviorally relevant dose of cannabis vapor that stimulated feeding in mice. To do this, male C57BL/6J mice were individually housed (*n* = 5) in an environmentally controlled vivarium and habituated to cannabis vapor chambers prior to any experimental manipulations. All mice had ad libitum access to standard rodent chow and water. On test days, mice were food restricted 6 h prior to any experimental manipulation. On day 1, all mice were placed in a vapor chamber filled with air for 5 min to serve as “air-treated” controls. Following air exposure, mice were returned to their respective home cages and chow was returned. Food intake was measured at the 2 h timepoint. Following 1 day of treatment recovery, mice were exposed to 100 mg of cannabis vapor over a 5-min period and subsequently returned to their home cages, as described previously. This experimental design was repeated to test food consummatory behavior at 200 mg and 400 mg doses of cannabis, respectively.

#### Experiment 5: In vivo calcium imaging of MBH neurons of mice exposed to cannabis vapor

To assess MBH neuronal activation in response to HFD following cannabis exposure, we completed in vivo calcium imaging experiments in freely behaving mice using the Inscopix nVista system. Briefly, 6–8 week old C57BL/6J mice (*n* = 4) maintained on a reverse 12/12 light cycle were anesthetized with isoflurane and unilaterally injected (AP =  − 1.7, ML =  +/− 0.3, DV = , 5.60 mm to 5.75) with 1.5 μl of pAAV.Syn.GCaMP6s.WPRE.SV40 (Addgene, #100843). Following the viral injection, a stereotaxic support arm was used to implant a graded index lens (Inscopix, 1050-002212) 200um above the viral injection site. Lenses were secured in place with C&B Metabond Quick Adhesive Cement System (Parkell, S380) and covered with clear Kwik-Sil silicone elastomer (World Precision Instruments, KWIK-SIL). After 4–5 weeks of viral incubation, mice were briefly anesthetized with isoflurane to allow for the placement of a base plate (Inscopix, 100-004095), which was positioned to ensure optimal clarity. A base-plate cover was fitted over the baseplate to ensure lens protection while not in use.

Two days following the base-plating procedure, mice were acclimated to the cannabis vapor chambers and the open field environment (Med Associates Inc., ENV-510). Prior to each session of open field acclimation, mice were acclimated to the vapor chambers (air only) for a 10-min session. Then, mice were placed in an open field environment fitted with an EthoVision XT system (Noldus, EthoVision XT 11.5) to record locomotor activity for a total of 20 min. For the first 10 min, no stimuli were presented in the environment. During the second 10 min, a small pellet of HFD was placed in the upper right corner. Mice were exposed to HFD in their home cages prior to open field acclimation to reduce neophobia. On day 1 of acclimation, mice were untethered in the open field environment. On day 2, mice were tethered to a dummy Inscopix imaging scope. Tethered acclimation sessions continued for 3–4 days until the mice were fully acclimated to the dummy scope placement.

On all test days, mice were food restricted for a total of 6.5 h beginning 3 h before lights off. On day 1, mice received air treatment for 5 min in the cannabis vapor chambers. 30 min after air exposure, mice were tethered to an Inscopix nVista miniscope and placed in the open field environment for 10 min without food and 10 min with HFD. EthoVision XT recordings occurred for the duration of the session. On day 2, mice received 200 mg cannabis over a 5-min period in the vapor chambers and the open field test described on day 1 was completed identically. At the conclusion of these experiments, all mice were euthanized, and lens placement verified.

#### Experiment 6: WIN effects on AgRP neuronal GABAergic sIPSCs.

To investigate the role of CB1R signaling on AgRP neurons, we performed uni- or bi-lateral pAAV.Syn.Flex.GCaMP6s.WPRE.SV40 (Addgene, #100845) ARC injections in 6–8 week old *Agrp*^*tm1(cre)Low1*^*/J* mice for cell-type specific electrophysiological recordings (Please see supplemental methods for full details of solution composition). Following 2 weeks of viral incubation, coronal slices (225 μm in the range- coronal plates 26–36; Bregma − 2.12 mm − 4.52 mm) were prepared for recording. AgRP cells were visually identified and voltage-clamped (Vh = − 60 mV). Internal solution was pH-adjusted to 7.2–7.3 with CsOH. All aCSF contained 2 mM kynurenic acid (a broad spectrum glutamate receptor blocker) to isolate spontaneous inhibitory post synaptic currents (sIPSCs) in the recording. Drugs (WIN 55,212-2; Cayman Chemical, #10009023) were dissolved in aCSF + kynurenic acid, bubbled with 95% O2/5% CO2 gas before being administered. All experimental conditions were conducted for 5 min each, with the baseline condition being conducted for at least 5 min, or until a stable baseline was reached. sIPSCs from the final 2 min of recording in each experimental condition were analyzed using Clampfit (Molecular Probes), using the function threshold search of event detection, using an amplitude threshold of 3 times the peak-to-peak amplitude of the noise, and then events were individually inspected with a further inclusion criterion of having a rise time at least 3× faster than the decay time. Average frequency was determined including temporally overlapping events, then all non-overlapping events were averaged to calculate mean amplitude. Detailed methods can be found in the supplemental information.

#### Experiment 7: chemogenetic inhibition of AgRP neurons

To determine if AgRP neurons regulate cannabis-induced feeding behavior, mice were injected with a DREADD virus specifically targeting the AgRP neurons in the MBH. Briefly, (*n* = 5) 6–8 week old *Agrp*^*tm1(cre)Low1*^*/J* mice (*n* = 5) maintained on a reverse 12/12 light cycle were anesthetized with isoflurane and bilaterally injected (AP =  − 1.7, ML =  +/− 0.3, DV = , 5.60 mm to 5.75) with 1.5 μl of pAAV.hSyn.DIO.hM4D(Gi).mCherry (Addgene, #44362). After 3–4 weeks of viral incubation, mice were acclimated to the vapor chambers for 5 min a day over 5 days with no manipulations to reduce neophobia. On test day 1, mice were injected with saline and immediately placed into the vapor chamber and exposed to air. On test day 2, mice were injected with saline and immediately exposed to 200 mg of vaporized cannabis (10.1% THC, 0.5% CBD, NIDA, Research Triangle, NC) for 5 min. This was repeated on test day 3, except here, mice received a 2 mg/kg dose of clozapine-N-oxide (CNO) immediately preceding vapor cannabis exposure and food intake was measured at 0.5, 1 and 2 h following vapor cannabis exposure. One week after the completion of the experiment, mice were transcardially perfused and the brains removed for histological verification.

### Statistical analyses

Food Intake, feeding microstructure, Bussey Touch Screen System (BTSS) activity, locomotor activity, in vivo Ca^2+^ imaging data, and DREADD manipulations data were analyzed by two-way repeated measures ANOVA in IBM SPSS Statistics 29.0.0.0 (IBM Corp.). Overall average meal size and meal number statistics were analyzed by one-way ANOVA and both metabolic and dose response data were analyzed by one-way repeated measures ANOVA in IBM SPSS Statistics 29.0.0.0. Tukey HSD was used for post-hoc analysis in all datasets except for locomotor activity data due to the unequal sample sizes. Graphs were subsequently generated in Graphpad Prism 9.0.0 (Graphpad Inc.). Data for cell activity were graphed in Origin Lab (Origin Lab, MA). Patch clamp data were analyzed using a paired students t-test. For each group, the number of subjects as well as the mean differences are reported. All statistical comparisons were conducted at the 0.05α level.

## Results

### Cannabis increases food intake in free-feeding rats

**Figure 1:**
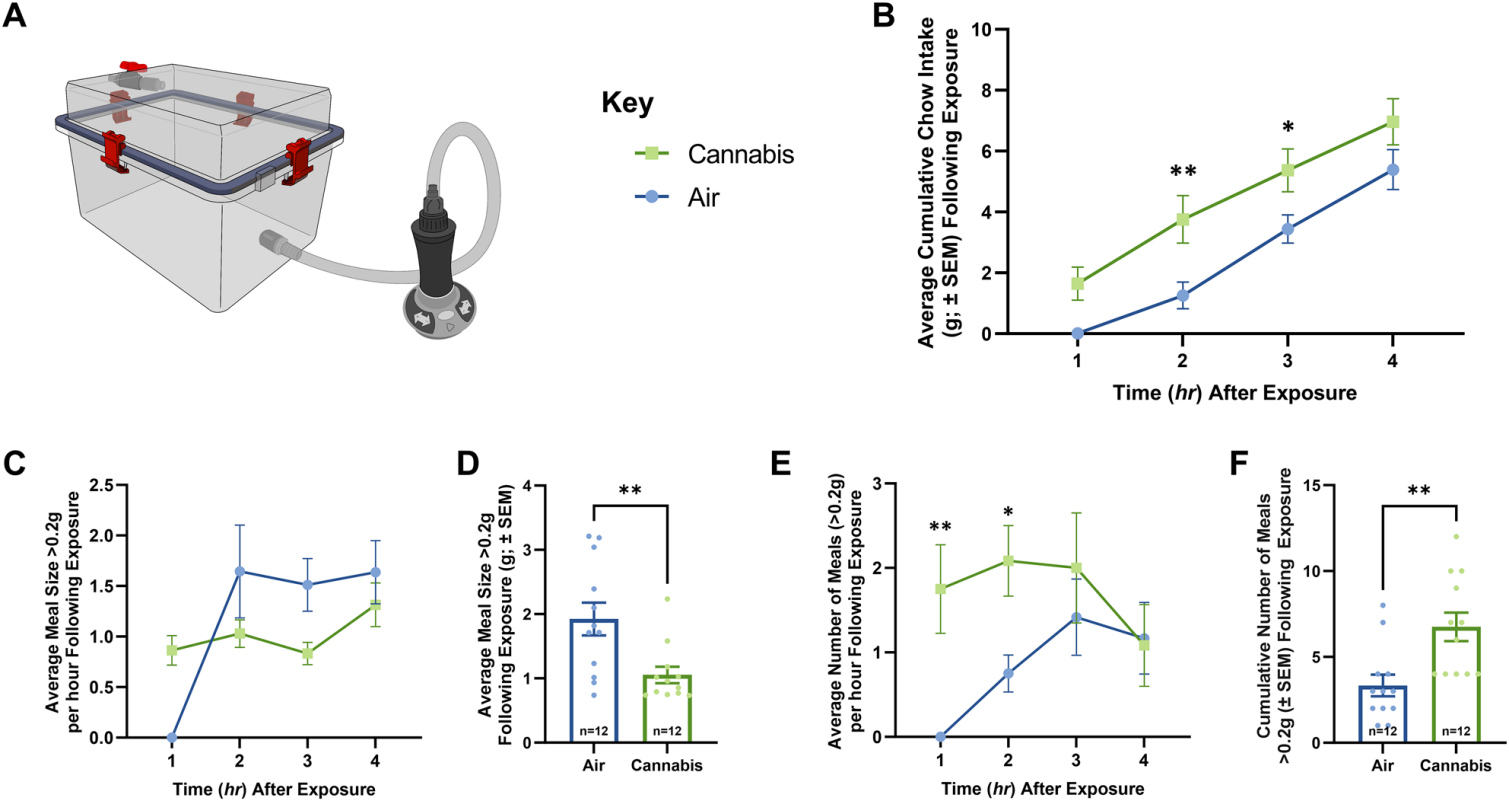
Vapor Cannabis Exposure Augments Meal Frequency. (**A**) Illustrates the vapor chamber apparatus used in the following studies. (**B**), (**C**), and (**E**) Represent quantification of ingested chow at 1-h intervals following air or cannabis exposure (*n* = 12/group), significance calculated with two-way repeated measures ANOVA with Tukey HSD post-hoc testing. (**D**) and (**F**) Represent quantification average and cumulative meal patterns (n = 12/group), significance calculated with one-way ANOVA test. (**B**) shows free-feeding rats exposed to 800 mg of cannabis consumed significantly more chow than air rats over 4 h. (**C**) Displays the average meal size per hour following cannabis and air exposure, statistical testing revealed a significance in the within subjects’ comparison. Meal pattern analysis revealed that vapor cannabis exposed rats consumed smaller meals relative to air-exposed controls (**D**), and that vapor cannabis exposure led to a trend toward increased total number of meals relative to air-exposed rats by hour (**E**), means comparison revealed significant differences at the 1 and 2 h timepoints and in total (**F**). See Supplemental Tables S1 and S2 for ANOVA testing results and post-hoc analysis results respectively.

To determine how cannabis vapor affects temporal feeding patterns, we housed rats in metabolic chambers with real-time automated feeding measurement of meal frequency and meal size following exposure to air or a behaviorally characterized dose of cannabis vapor known to elicit feeding behavior^18^ (800 mg; Fig. 1A). Relative to air-treated controls, exposure to cannabis vapor produced a transient, significant increase in food intake 2 and 3 h post exposure (Fig. 1B). Further analysis of meal patterns revealed that cannabis vapor exposure promoted increased meal frequency and reduced meal size throughout the evaluation period (Fig. 1C–F), suggesting that inhaled cannabis may provoke motivational components of feeding.

### Vapor cannabis regulates general locomotor activity in a food specific fashion

**Figure 2:**
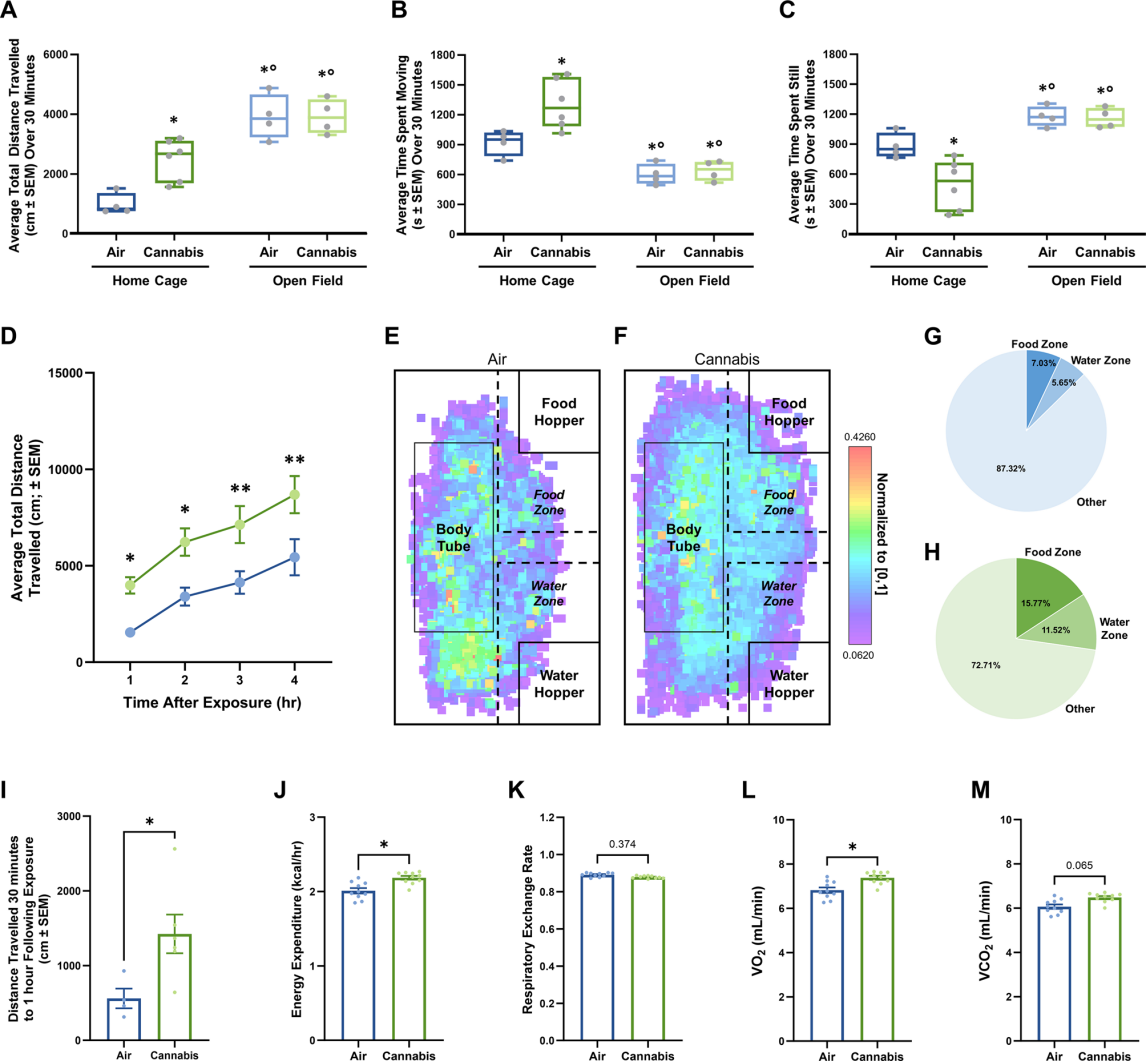
Vapor Cannabis Alters Locomotor Activity in a Food Specific Fashion. Two cohorts of rats were tested each with an air control and cannabis group, cohort 1 (*n* = 4/group) was placed into an open field 1 and 2 h after cannabis exposure, cohort 2 rats (*n* = 4 for air controls, and *n* = 6 for cannabis group) were housed and returned to their home cages in the Sable Promethion metabolic system. (**A**)–(**C**) Compares the locomotor data from both groups for the 30 min immediately after exposure and were analyzed with a two-way ANOVA and Tukey HSD post-hoc testing. (**A**)–(**C**) Key: * = significantly different than Home Cage Air rats, ○ = significantly different than Home Cage Cannabis rats. (**A**) Displays the total distance traveled by each group of rats, rats returned to their home cage traveled significantly less than rats placed in the open field overall and air exposed rats traveled significantly less than cannabis exposed rats overall. (**B**) Shows the differences in time spent moving with rats returned to their home cage spending significantly more time moving than rats in the open field and cannabis exposed rats moving significantly more than air exposed rats. (**C**) Displays the amount of time rats were not moving, rats returned to their home cage spent significantly less time not moving relative to the rats in the open field and air exposed rats spent significantly more time not moving than cannabis exposed rats overall. Post-hoc testing for distance, time spent moving, and time spent still revealed significant differences between all groups except for air exposed and cannabis exposed rats in the open field. Statistical results for (**A**)–(**D**) ANOVAs and pairwise comparisons are displayed in Supplemental Tables S3 and S4 respectively. (**D**)–(**M**) Displays data from rats housed only in the Sable Promethion system. (**D**) Reveals cannabis exposed rats in their home cage traveled significantly more than air exposed rats from 1 to 4 h after exposure; post-hoc testing revealed significant differences at each time point. (**E**)–(**F**) Illustrates the X- and Y- positions of the rats in the 1 h after air or cannabis exposure. There is a visible but not significant increase in the time spent near the food hopper in the cannabis exposed mice (**G**–**H**; *n* = *4* for air and *n* = *6* for 800 mg cannabis, unpaired two-tailed t-test Welch Corrected; t_(7.801)_ = 1.382, *P* = 0.2052). (I)–(**M**) Displays the metabolic data collected 30 min after exposure to 1 h after exposure in the second cohort of rats. (**I**) reveals cannabis exposed rats traveled significantly more than air exposed rats. In (**J**)–(**M**), data from each rat was binned into three-minute segments over 30 min, from 30 min to 1 h after exposure. (**J**) Shows a significant increase in energy expenditure (kcal/hr) in cannabis exposed rats, but no significant difference in respiratory exchange rate (**K**). Cannabis exposed rats had a significantly increased rate of oxygen consumption (VO_2_) relative to air rats (**L**), however (**M**) reveals no significant difference in rate of carbon dioxide emission (VCO_2_) levels between cannabis and air exposed rats. All metabolic analyses were conducted using two-way repeated measures ANOVA testing, data is shown in Supplement Table S5.

Tetrahydrocannabinol (THC), the primary psychoactive constituent of the cannabis plant, produces sedative effects in patients and rodents^23^. As a result, THC-based therapies are often not well tolerated in the clinical population^7,8,24,25^. Thus, to determine if the observed appetite stimulation induced by cannabis vapor was accompanied by potentially adverse sedative effects, we conducted an open field assay and collected data from rats housed in metabolic chambers to examine potential changes in locomotor activity (Fig. 2A–C). Rats exposed to cannabis vapor and placed in the open field arena without access to food did not display differences in total distance traveled (Fig. 2A; right), cumulative movement (Fig. 2B; right), or time spent still (Fig. 2C; right) relative to air-treated control rats. In contrast, when rats were exposed to cannabis in the presence of food in metabolic chambers, they traveled a greater total distance (Fig. 2A; left), displayed increased cumulative movement (Fig. 2B; left), spent less time still (Fig. 2C; left) and more time around the food hopper (Fig. 2E–H) relative to air-treated control rats. Notably, when compared to rats with no food present, both distance traveled and time spent still were significantly decreased in rats with food present, while time spent moving was significantly increased, indicating that in the presence of food, cannabis treated rats spent more time around the food hopper as opposed to other areas of the environment (Fig. 2A–C). Importantly, rats continued to display increased total distance traveled for up to 4 h after exposure (Fig. 2D). Together these data suggest that appetite stimulation with this dose (800 mg) of cannabis vapor does not induce noticeable sedative effects and instead, motivates behavior towards food.

### Cannabis augments metabolic activity

Previous studies suggest that inhaled cannabis leads to increases in resting metabolic and ventilation rate^26^. In the present study, rats exposed to an acute dose of vaporized cannabis displayed no significant difference in respiratory quotient (RQ), the ratio of carbon dioxide emission and oxygen consumption, compared to air controls (Fig. 2K). However, the rate of oxygen consumption was significantly increased in the cannabis exposed rats (Fig. 2L) while the rate of carbon dioxide emission did not reach statistical significance (Fig. 2M). Importantly, this ratio is often used as an indicator of the macronutrient utilization, a ratio of 0.7 indicates fat metabolism, whereas 1.0 indicates carbohydrates, and 0.8 suggests a mixed diet^21^. Both cannabis and air exposed rats had average ratios around 0.88 suggesting they are metabolizing both carbohydrates and fats (Fig. 2K). Interestingly, and in support of the current theory that cannabis use increases metabolism^26^, our data indicate that cannabis exposed rats had a significantly higher rate of energy expenditure (Fig. 2J), an observation that occurred during the same timeframe when rats were more active (Fig. 2I).

### Cannabis augments food-motivated behavior

**Figure 3:**
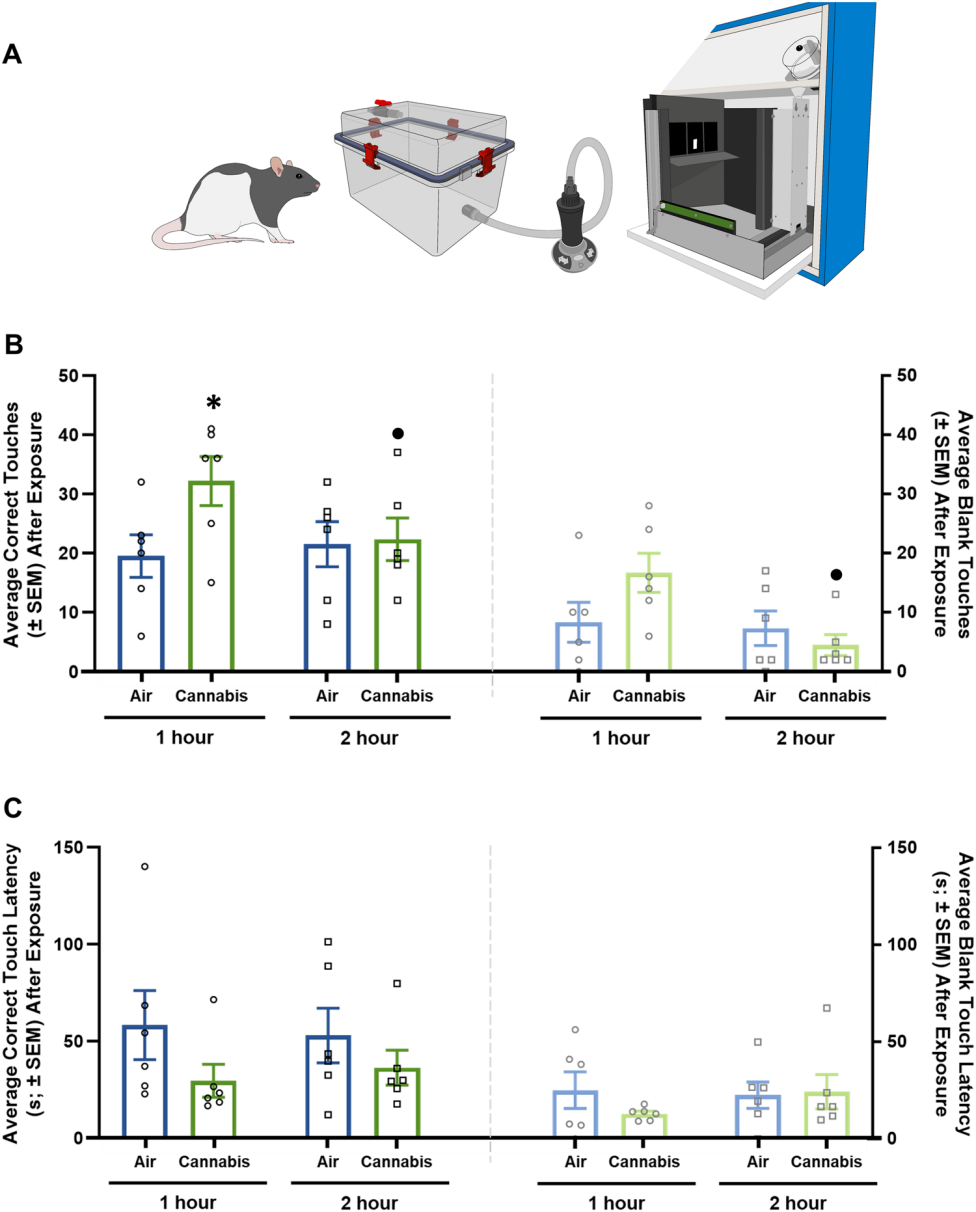
Vapor Cannabis Augments Food Seeking Behavior. (**A**) Illustrates the experimental design for operant responding studies in male Long Evans rats. Rats were exposed to air or cannabis on two days, one for 1-h post-exposure and one for 2-h post-exposure, statistical analysis was completed with two-way ANOVA repeated measures tests with Tukey HSD post-hoc testing. (**B**) illustrates correct and blank touches for air and cannabis exposed rats at both 1 and 2 h after exposure. The interaction between time and cannabis exposure for correct touches was significant (left side) but no significance was detected for time or air and cannabis exposure alone. Post-hoc testing revealed significant differences in number of correct touches between 1-h air and cannabis and cannabis exposure at 1 and 2-h timepoints. However, blank touches (**B**; right side) for both time and the interaction of cannabis or air and time were significantly different. Post-hoc testing of blank touches revealed a significant difference between cannabis exposure at 1 and 2-h timepoints. (**C**) Illustrates touch latency for both correct (left) and blank touches (right). No significant differences were detected in touch latency. Statistical Analysis Results of two-way repeated measures ANOVA testing and post-hoc Tukey HSD testing are shown in Supplemental Tables S6 and S7 respectively. Key: * = significantly different than 1 h Air exposed rats, • = significantly different than 1 h Cannabis exposed rats.

To determine if cannabis sativa enhances the motivational properties of food, we evaluated operant performance for sucrose in rats exposed to cannabis vapor (Fig. 3A). Vapor cannabis exposure caused a significant increase in operant responding for sucrose 1 h after exposure (Fig. 3B; left) without affecting the total number of blank touches, or the latencies to perform correct or blank touches (Fig. 3B, C). In contrast, no differences in operant responding for sucrose were detected when rats were tested 2 h following vapor cannabis exposure (Fig. 3B, C). However, cannabis exposed rats did have significantly less correct and blank touches 2 h after exposure relative to 1 h after, indicating that cannabis augments food-motivated behavior in a time-dependent fashion.

### Vapor cannabis stimulates food intake in mice

**Figure 4:**
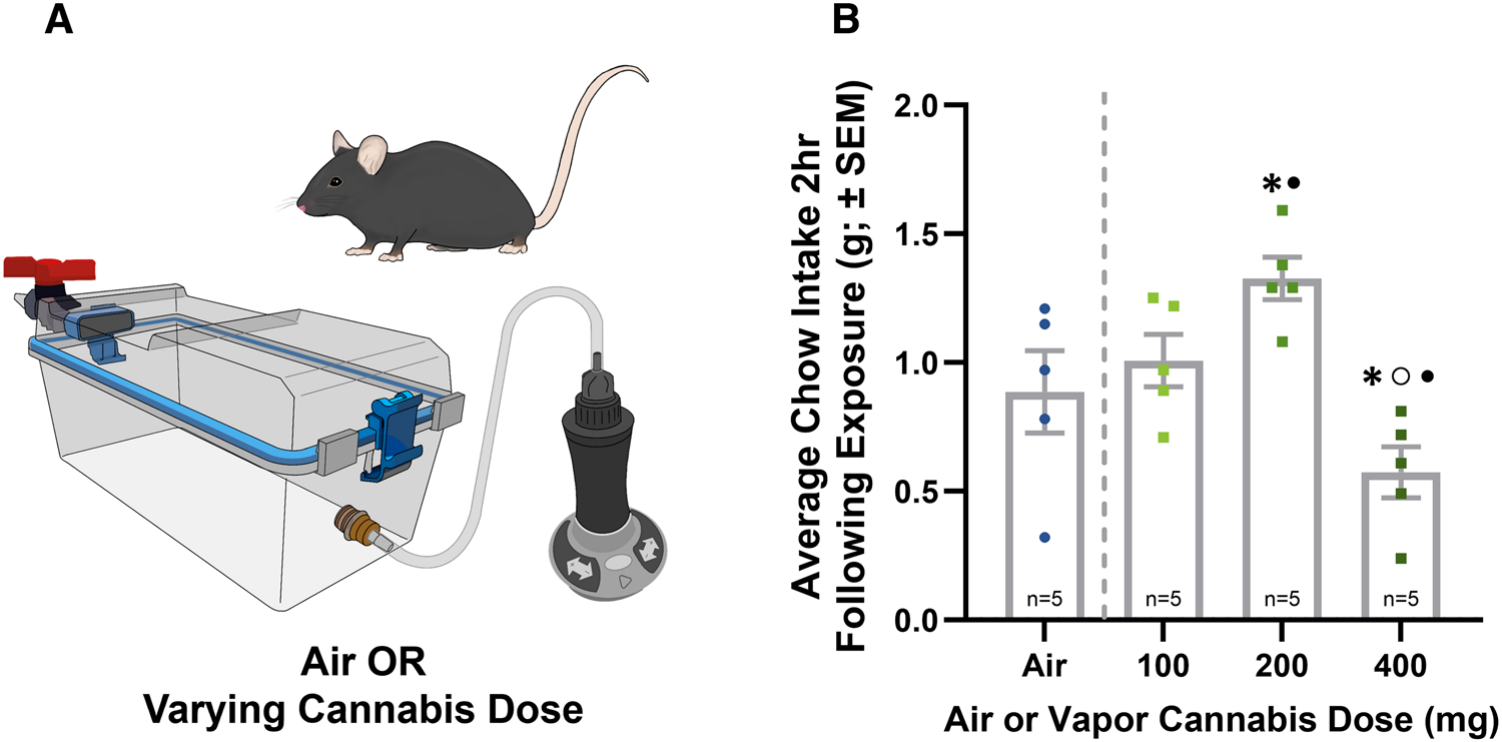
C57BL/6J Cannabis Dose Response in C57BL6J Mice. (**A**) Illustrates the experimental design of the dose response in male C57BL6J mice. (**B**) Depicts the cannabis feeding dose response in *n* = 5 C57BL/6J mice 2 h following 5 min of vapor cannabis exposure. The dose response model between doses at the same timepoint was significantly different (One-Way repeated measures ANOVA, F_(3,12)_ = 14.065, ***P = 0.0003). Post-hoc testing (Tukey HSD) revealed a significant increase in food intake at 200 mg cannabis. Significant differences were also seen between 200 and 400 mg, as well as 100 mg and 400 mg. See Supplemental Table S8 for detailed statistical results. Key: * = significantly different than Air exposure, • = significantly different than 100 mg exposure, ○ = significantly different than 200 mg exposure.

To conduct in vivo imaging experiments, we first needed to establish a dose response for cannabis-induced feeding behavior in mice (Fig. 4A). Our data indicate that food intake in C57BL6J mice exposed to the 100 mg dose did not differ significantly from air. However, mice displayed a significant increase in food intake when exposed to the 200 mg cannabis dose whereas a significant decrease was detected at the 400 mg dose (Fig. 4B). It is important to note here that this dose (200 mg), was 4× less than that required for cannabis-induced appetite stimulation in rats and followed a similar temporal pattern as previously published in this model^18^. Subsequently we used this dose in all calcium imaging experiments and chemogenetic manipulations.

### In vivo Ca^2+^ imaging in mixed populations of MBH neurons

**Figure 5:**
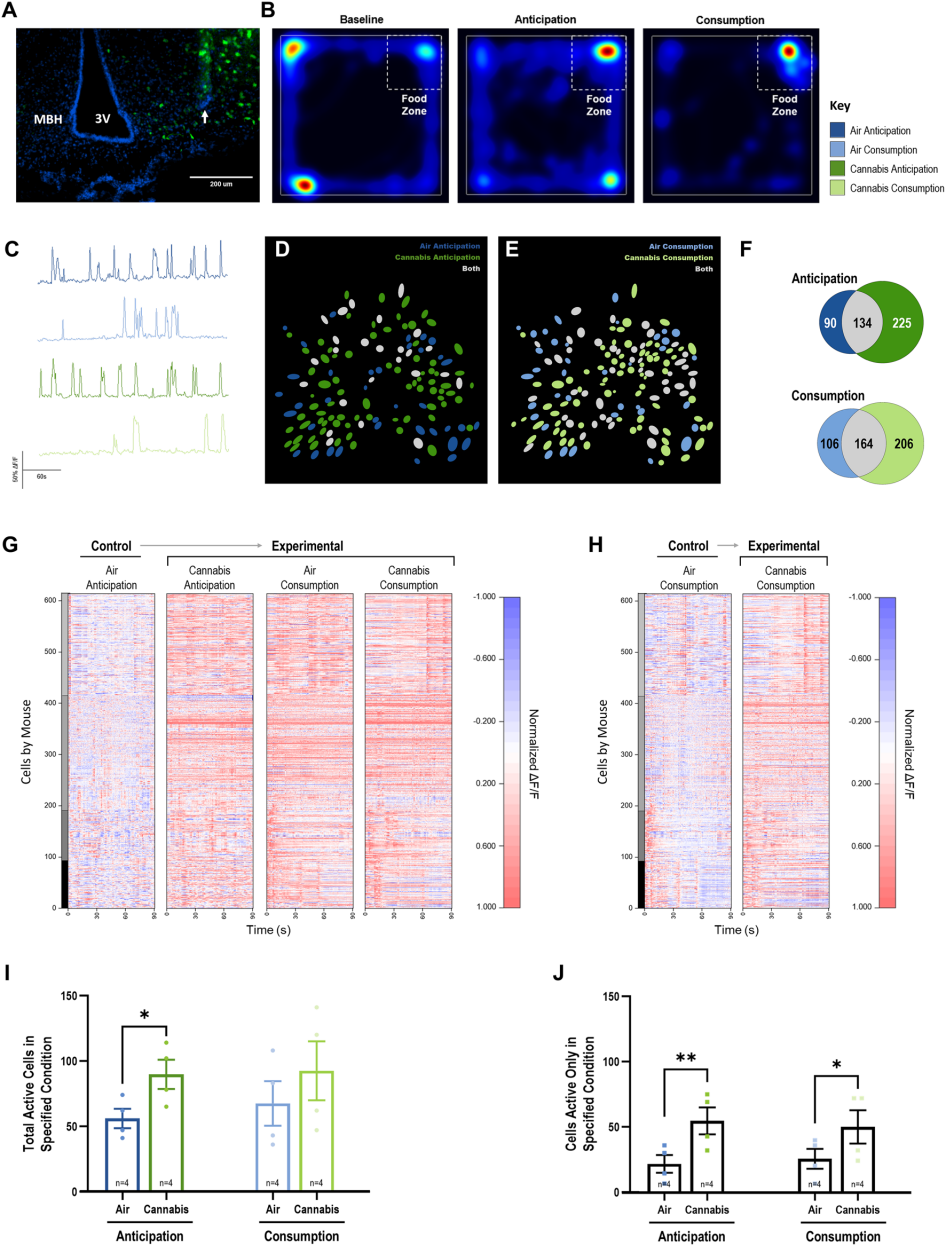
Cannabis Activates a Distinct Population of MBH Neurons. (**A**) illustrates GFP (green) and DAPI (blue) labeled neurons in the MBH of C57BL6/J male mice (scale bar set at 200 μm). The white arrow denotes the GRIN lens track. (**B**) Heatmap illustrating conditioned approach behavior within the arena where HFD was offered. (**C**) Representative DF/F Ca^2+^ traces from MBH neurons in mice exposed to air (blue) or cannabis (green). (**D**)–(**E**) field of view illustrating non-overlapping, overlapping and novel neurons activated by cannabis during HFD anticipation (**D**) and consumption (**E**). Blue color = air condition, white = overlapping neurons, green = cannabis condition. (**F**) Venn diagrams quantifying all neurons active in both air and cannabis conditions during anticipation (top) or consumption (bottom) in gray, and each condition independently (air = blue, green = cannabis). (**G**) Normalized DF/F Z-score heatmapping of neuronal activation in each condition relative to the air anticipation baseline over a 90 s period for each mouse (*n* = 4; each mouse cell set is indicated in separate shades of gray on the y-axis) included in the analysis. (**H**) Normalized DF/F Z-score heatmapping of neuronal activation in cannabis consumption relative to the air consumption baseline over a 90 s period for each mouse. Active cells differences were analyzed using a two-way repeated measures ANOVA with Tukey HSD post-hoc testing. (**I**) Quantification of active neurons in each condition, both air versus cannabis and the interaction of air versus cannabis and feeding stage, anticipation versus consumption, were significantly different. Post-hoc analysis revealed overall significance between air and cannabis totals, and of anticipation (left) for air and cannabis. (**J**) Quantification of activated MBH cells when mice entered the FZ. Both air versus cannabis and the interaction between air and cannabis and anticipation and consumption was significantly different. Post-hoc testing revealed significant differences between air and cannabis exposure in both anticipation and consumption. See Supplemental Tables S9 and S10 for detailed statistical information for the two-way repeated measures ANOVA testing, and the post-hoc Tukey HSD testing respectively.

The MBH, regulates appetite and contains neurons that express the cannabinoid-1 receptor (CB1R; 17). Thus, we reasoned neurons in this region may participate in cannabis-induced appetite. To understand how MBH neuronal activity was modified by cannabis, we applied in vivo microendoscopic imaging to visualize somatic intracellular calcium [Ca^2+^] dynamics in mixed neuronal ensembles in freely behaving mice. Specifically, we expressed the calcium sensor GCamp6s in the MBH, specifically the ARC, of male C57BL/6J mice and then utilized microendoscopes to visualize changes in [Ca^2+^] in neurons when mice approached or consumed palatable food (Fig. 5A). Mice exposed to this paradigm rapidly learned to approach the food containing zone (FZ), where a palatable high fat diet (HFD) was consistently offered (Fig. 5B). Using this approach, our first experiment involved exposing mice to air only in the vapor chambers (control) then visualizing GCamp6s fluorescence, which reflects changes in intracellular Ca^2+^ dynamics (Fig. 5C, G, H). Cannabis-exposed mice displayed increased [Ca^2+^] in numerous MBH neurons that became activated when mice entered the FZ (Fig. 5I). In agreement with previous reports^27,28^, a portion of FZ activated neurons remained active when HFD was consumed whereas others were inhibited. In addition, a separate population of neurons displayed increased [Ca^2+^] only when HFD was consumed (Fig. 5J).

### Vapor cannabis stimulates food anticipatory responses in MBH neurons

We next exposed the same mice to the behaviorally characterized dose of cannabis shown to increase feeding in mice (200 mg, Fig. 4B) prior to placement in the test arena. Importantly, the number of active MBH neurons remained nearly constant during both the anticipatory and consummatory timeframes following cannabis exposure (Fig. 5I). Under this condition, we observed a significant increase in the number of MBH neurons that displayed [Ca^2+^] when mice entered the FZ relative to air FZ active neurons (Fig. 5F, J). A similar effect was observed once HFD was delivered (Fig. 5F, J). Remarkably, we observed increased [Ca^2+^] in a novel population of FZ and HFD stimulated MBH neurons in mice exposed to cannabis (Fig. 5D, E, J, supplemental video file S1). Overall, these observations indicate that cannabis exposure augments activity of MBH neurons during functionally diverse aspects of feeding behavior.

**Figure 6:**
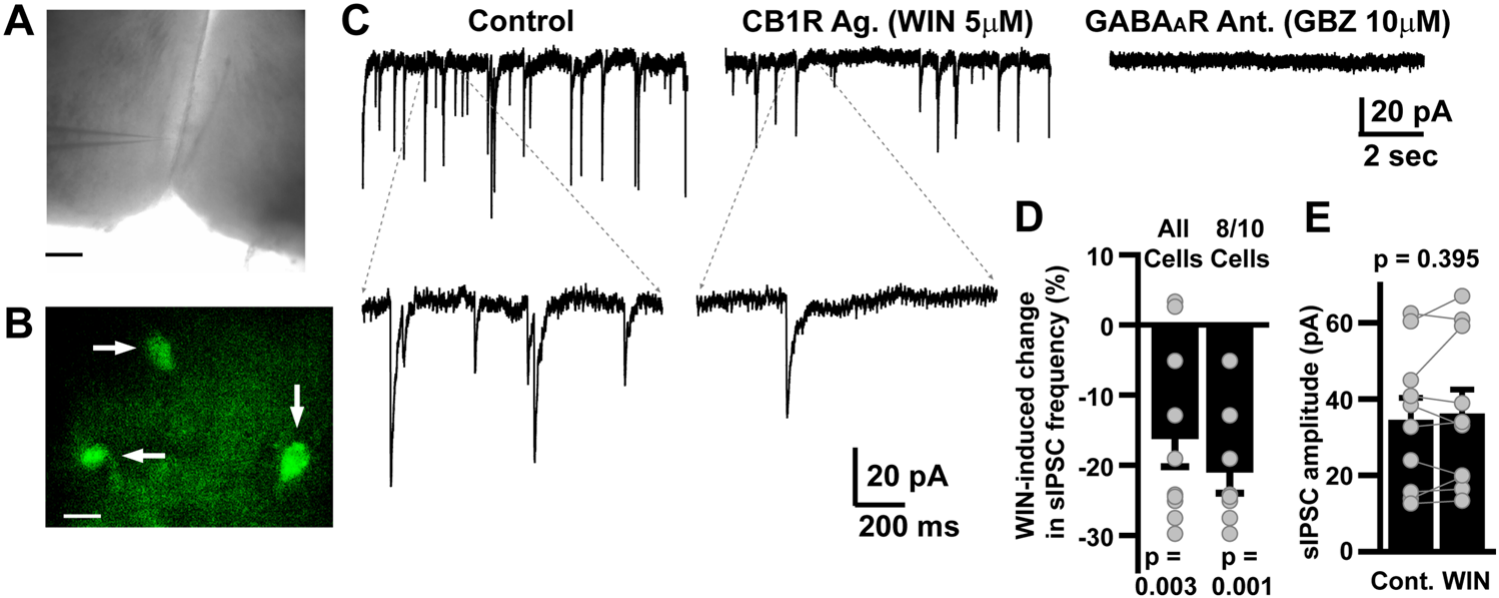
CB1R Agonist WIN Reduces AgRP Neuron sIPSC Frequency. (**A**) Illustrates a DIC image of a coronal slice (200 mm) of hypothalamus with a recording electrode located in the Arcuate Nucleus. Scale Bar = 250 mm. Fluorescence image example of eGFP labelled AgRP neurons, from which all recordings were made (**B**; scale bar = 25 mm). (**C**) Demonstrates a representative voltage-clamp recording (V_h_ = -60 mV, and E_Cl_- = 0 mV making GABA_A_R currents inward/downward) from an eGFP labelled AgRP neuron under control conditions (ACSF, top left panel), in the presence of CB1R agonist (WIN, 5 mM, top middle panel), and in the presence of the GABA_A_R antagonist (GABAzine, 10 mM, top right panel). Bottom panels in left and middle panels are expanded time scale specimen recordings taken from the region in the top panels indicated by dashed lines. (**D**) illustrates a bar chart and overlaid dot plots of percent change in sIPSC frequency induced by WIN for all cells (left bar) or for only those cells that displayed a suppression of frequency by WIN (8/10, right bar). In both cases, WIN caused a significant suppression of sIPSC frequency, as assessed by a one sample Students t-test, assuming a population mean of 0% change (*P* = 0.003 and 0.001 respectively). WIN did not significantly affect the amplitude of AgRP neuron sIPSCs (*P* = 0.395; **E**).

### CB1R activation disinhibits AgRP neurons

Within the MBH, AgRP neurons are instrumental in promoting hunger and motivation to obtain food^19^. In addition, recent reports indicate that AgRP neurons receive presumed GABAergic afferent inputs that express CB1Rs^20^. With this in mind, we employed an ex vivo slice electrophysiology approach to determine if activation of CB1Rs affected GABAergic synaptic activity onto AgRP neurons. To accomplish this, we injected *Agrp*^*tm1(cre)Low1*^*/J* mice with an AAV expressing a GFP reporter to allow visualization and selection of AgRP neurons for patch-clamp recording (Fig. 6A, B). Recordings were performed in the presence of a glutamate receptor antagonist (Kynurenate, 1 mM) to isolate GABA_A_ receptor (GABA_A_R)-mediated synaptic currents. Voltage clamped (Vh = − 60 mV, with E_Cl-_ =  ~ 0 mV, making GABAergic currents inward) AgRP neurons exhibited robust spontaneous inhibitory post synaptic currents (sIPSCs), mediated by GABA_A_Rs, as evidenced by their elimination following treatment with the GABA_A_R antagonist GABAzine (10 mM; Fig. 6C). Bath application of the selective CB1R agonist, WIN 55, 212-2 significantly reduced the frequency of sIPSCs onto AgRP neurons (Figs. 6C, D) without altering peak amplitude (Fig. 6E). These data confirm that functional CB1Rs are indeed expressed on GABAergic synapses onto MBH neurons, and that their activation suppresses vesicular release of GABA, resulting in disinhibition of AgRP neurons.

**Figure 7:**
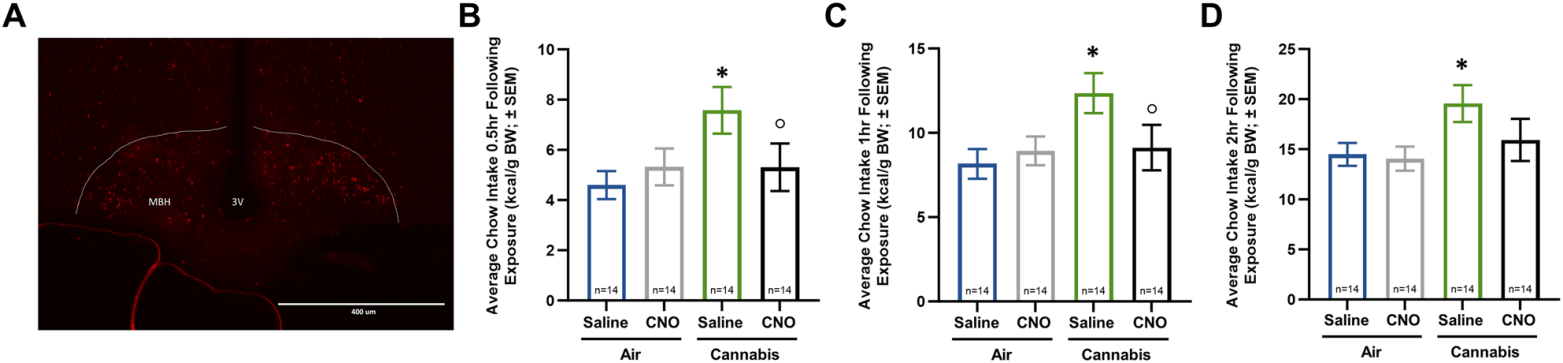
Chemogenetic Inhibition of AgRP Neurons Attenuates Cannabis-Induced Feeding Behavior. (**A**) indicates mCherry (red) labeled neurons located in the MBH of the hypothalamus. Scale bar = 400 μm). (**B**)–(**D**) depict differences between air and cannabis and saline and CNO injections (*n* = 14) in DREADD infected mice at 30 min (**B**), 1 h (**C**), and 2 h (**D**). Two-way repeated measures ANOVA testing revealed significant differences in air versus cannabis exposure overall and the interaction between air and cannabis and saline and CNO at 30 min, and one hour, but only between overall air and cannabis exposure at 2 h. Tukey HSD post-hoc testing at both 30 min and 1 h revealed significant differences between air and cannabis with saline injections, and cannabis with saline and CNO. Post-hoc testing at two hours revealed significance between air and cannabis with saline injections. See Supplemental Tables S11 and S12 for detailed statistical results. Key: * = significantly different than air saline exposure, ○ = significantly different than cannabis saline exposure.

### AgRP neurons regulate the appetite stimulatory properties of cannabis sativa

Recent work indicates that pro-opiomelanocortin (POMC) neurons regulate feeding induced by endocannabinoids^29,30^. However, it is less clear if appetite-stimulatory AgRP neurons contribute to this process. In the present study chemogenetic inhibition of AgRP neurons attenuated the appetite stimulatory properties of vaporized *cannabis sativa*. Specifically, *Agrp*^*tm1(cre)Low1*^*/J* mice expressing inhibitory DREADD receptors (hM4D(Gi) (Fig. 7A) displayed attenuated intake of chow following cannabis exposure when treated with CNO relative to saline injected controls (Fig. 7B–D). These data indicate that AgRP neurons are functionally relevant for regulation of cannabis-induced feeding behavior.

## Discussion

The appetite promoting effects of *cannabis sativa* have been recognized for centuries^31–34^, however, surprisingly, the biological mechanisms that underlie this process have remained largely unknown. In this regard, our data demonstrate that inhalation of cannabis vapor augments the appetitive phases of feeding behavior as evidenced by an increase in the number of meals consumed, a decrease in meal size and enhanced effort-based responding for palatable food. Notably, these behavioral observations occurred in the absence of reduced locomotor activity, and in the presence of increased energy expenditure.

The CB1R is expressed within the MBH of both rodents^35^ and humans^36,37^. In rodents, CB1R mRNA is expressed within a novel population of n19.Gpr50^+^ neurons^35^ as well as kisspeptin neurons^33^. In humans, CB1R mRNA is expressed in pro-opiomelanocortin (POMC) neurons^39^, an observation in agreement with previous rodent studies which indicate that endocannabinoids target POMC neurons to stimulate food intake^29,30^. The ubiquitous expression pattern of CB1R suggests that inhaled cannabis may alter the activity of multiple populations of MBH neurons. Our in vivo Ca^2+^ imaging data indicate that vapor cannabis increases activity of distinct populations of temporally defined MBH neurons. Specifically, we find that mice exposed to cannabis vapor display increased activity within MBH neurons that encode meal anticipation or meal consumption. These data are the first to detail the in vivo effects of inhaled cannabis on neuronal activity in an appetite-regulatory region of the CNS and suggest that MBH neurons may play a strong regulatory role for feeding behaviors stimulated by cannabis drugs.

Importantly, clinical evidence suggests that maladaptive reductions in hypothalamic CB1R expression may contribute to metabolic disturbances^37^. Thus, understanding the neurobiological mechanisms stimulated by cannabis exposure is an essential step for development and refinement of translational therapies for metabolic disease. In this context, our data indicate that GABAergic afferent terminals contain CB1Rs that exert a strong inhibitory tone on AgRP neurons. Moreover, we find that activation of GABAergic afferent CB1Rs significantly reduced the frequency of sIPSCs in AgRP neurons, without affecting their amplitude. These data suggest that activation of CB1Rs directly suppresses vesicular release of inhibitory GABA onto AgRP neurons, raising the possibility that AgRP neurons can be disinhibited by cannabinoid drugs to promote appetite. In support of this notion, we find that the appetite stimulatory properties of cannabis are reduced in mice following chemogenetic inhibition of AgRP neurons. It is important to note here that cannabis-induced feeding was not entirely ameliorated after AgRP inhibition, thus our studies do not rule out the contribution of separate CNS regions or additional signaling mechanisms (cannabinoid-2 receptor, ion channels, or novel G-coupled protein receptors) as important regulators of cannabis-induced feeding behavior. That said, the data presented here provide new mechanistic insights regarding the neurobiological mechanisms involved in cannabis-induced appetite and support the hypothesis that cannabinoid-CB1R interaction within MBH neurons contribute to the appetite-stimulatory properties of inhaled cannabis.

### Supplementary Information

Supplementary Tables.

Supplementary Information.

Supplementary Video 1.

## Data Availability

All data are available in the main text or the supplementary materials.
