# Supplementary material for: Cannabis Sativa targets mediobasal hypothalamic neurons to stimulate appetite
Source: Sci Rep. 2023 Dec 27;13:22970. doi: 10.1038/s41598-023-50112-5 (PMC10752887; doi:10.1038/s41598-023-50112-5)
Supplement: Supplementary file 1 — Supplementary Tables. [file 41598_2023_50112_MOESM1_ESM.docx]

**Supplemental Data**

***Cannabis Sativa* Targets Mediobasal Hypothalamic Neurons to Stimulate Appetite.**

Emma C. Wheeler^1^, Pique Choi^1^, Joanne De Howitt^1^, Sumeen Gill^1^, Shane Watson^1^, Sue Yu^1^, Peyton Wahl^1^_,_ Cecilia Diaz^1^, Claudia Mohr^1^, Amy Zinski^2^, Zhihua Jiang^2^, David Rossi^1^, Jon F. Davis^1^

^1.^ Department of Integrative Physiology and Neuroscience, Washington State University, Pullman, WA, USA.

^2.^ Department of Animal Sciences, Washington State University, Pullman, WA, USA.

**Abbreviated title**: MBH Neurons Regulate Cannabis-Induced Feeding

Correspondence should be addressed to:

Jon F. Davis, PhD

Veterinary Biomedical Research Building, Room 115

Washington State University

Pullman, WA 99164

509-335-8163

Jon.davis@wsu.edu

Funding: State of Washington Dedicated Marijuana Account (dMAc) grant from the Alcohol and Drug Abuse Research Program (ADARP) grant number 130625–003 to JFD, and NIAAA grant R01AA026078 to DJR.

Disclosures: The authors declare no conflict of interest

Keywords: Cannabis, AgRP, Feeding

*Wheeler et al. – Data Supplement MBH Neurons Regulate Cannabis-Induced Feeding*

**Supplemental Table S1. ANOVA Analysis of meal patterns of air and cannabis exposed rats.**

| ***Source*** | | ***F-Statistic*** | ***p-value*** | ***η_p_^2^*** |
| --- | --- | --- | --- | --- |
| *Average Chow Intake ††* | |  |  |  |
|  | Time (1hr-4hr) | 64.474 | **<0.0001** | 0.746 |
|  | Drug (Air v. Cannabis) | 7.038 | **0.015** | 0.242 |
|  | Time*Drug | 0.542 | 0.655 | 0.024 |
|  |  |  |  |  |
| *Meal Size Over Time ††* | |  |  |  |
|  | Time (1hr-4hr) | 3.436 | 0.038 | 0.135 |
|  | Drug (Air v. Cannabis) | 0.075 | 0.787 | 0.003 |
|  | Time*Drug | 1.062 | 0.358 | 0.046 |
|  |  |  |  |  |
| *Average Meal Size †* | | 9.375 | **0.006** | 0.299 |
|  |  |  |  |  |
| *Average Meal Number over Time ††* | |  |  |  |
|  | Time (1hr-4hr) | 1.248 | 0.299 | 0.054 |
|  | Drug (Air v. Cannabis) | 11.181 | **0.003** | 0.337 |
|  | Time*Drug | 1.582 | 0.202 | 0.067 |
|  |  |  |  |  |
| *Cumulative Meal Number †* | | 11.050 | **0.003** | 0.334 |

*n=12/group. † One-Way ANOVA. †† Two-Way ANOVA with Repeated Measures Means Comparisons; Tukey HSD, α = 0.05. η_p_^2^: partial eta squared effect size. Degrees of Freedom: Time(3), Interaction(3), Error Within Subjects (66), Drug(1), Error Between Subjects(22).*

*Wheeler et al. – Data Supplement MBH Neurons Regulate Cannabis-Induced Feeding*

**Supplemental Table S2.** **Tukey HSD means comparison of meal patterns in air and cannabis exposed rats.**

| ***Measurement*** | | | ***Air***  *Mean ± SEM* | ***Cannabis***  *Mean ± SEM* | ***Mean Difference***  *(95% CI)* | ***q-value*** | ***p-value*** |
| --- | --- | --- | --- | --- | --- | --- | --- |
| *Chow Intake ††* | | |  |  |  |  |  |
|  | *Air v. Cannabis (Overall)* | | 2.526 ± 0.306 | 4.433 ± 0.650 | 1.907 (0.416 to 3.398) | 3.752 | **0.015** |
|  | *Drug*Time* | |  |  |  |  |  |
|  |  | 1 hour | 0.015 ± 0.009 | 1.644 ± 0.568 | 1.629 (-0.119 to 3.377) | 2.631 | 0.067 |
|  |  | 2 hours | 1.258 ± 0.457 | 3.757 ± 0.815 | 2.499 (0.751 to 4.247) | 4.037 | **0.006** |
|  |  | 3 hours | 3.441 ± 0.485 | 5.370 ± 0.736 | 1.929 (0.181 to 3.677) | 3.115 | **0.031** |
|  |  | 4 hours | 5.391 ± 0.682 | 6.962 ± 0.793 | 1.571 (-0.177 to 3.319) | 2.538 | 0.077 |
|  |  |  |  |  |  |  |  |
| *Meal Size Over Time ††* | | |  |  |  |  |  |
|  | *Air v. Cannabis (Overall)* | | 0.842 ± 0.359 | 0.793 ± 0.129 | -0.049 (-0.420 to 0.322) | 0.387 | 0.787 |
|  | *Drug*Time* | |  |  |  |  |  |
|  |  | 1 hour | 0.000 ± 0.000 | 0.519 ± 0.138 | 0.519 (-0.294 to 1.332) | 1.802 | 0.207 |
|  |  | 2 hours | 1.018 ± 0.417 | 1.081 ± 0.250 | 0.062 (-0.751 to 0.876) | 0.217 | 0.879 |
|  |  | 3 hours | 1.049 ± 0.334 | 0.620 ± 0.173 | -0.429 (-1.242 to 0.384) | 1.489 | 0.296 |
|  |  | 4 hours | 1.300 ± 0.419 | 0.951 ± 0.302 | -0.348 (-1.162 to 0.465) | 1.210 | 0.395 |
|  |  |  |  |  |  |  |  |
| *Cumulative Meal Number Over Time ††* | | | |  |  |  |  |
|  | *Air v. Cannabis (Overall)* | | 0.833 ± 0.354 | 1.729 ± 0.216 | 0.896 (0.340 to 1.451) | 4.729 | **0.003** |
|  | *Drug*Time* | |  |  |  |  |  |
|  |  | 1 hour | 0.000 ± 0.000 | 1.750 ± 0.524 | 1.750 (0.512 to 2.988) | 3.992 | **0.006** |
|  |  | 2 hours | 0.750 ± 0.218 | 2.083 ± 0.417 | 1.333 (0.096 to 2.571) | 3.042 | **0.035** |
|  |  | 3 hours | 1.417 ± 0.452 | 2.000 ± 0.651 | 0.583 (-0.654 to 1.821) | 1.331 | 0.350 |
|  |  | 4 hours | 1.167 ± 0.423 | 1.083 ± 0.484 | -0.083 (-1.321 to 1.154) | 0.190 | 0.893 |

*n=12/group. †† Two-Way ANOVA with Repeated Measures Means Comparisons, Tukey HSD* *α = 0.05. Degrees of Freedom: Drug(22), Interaction(66/per timepoint).*

*Wheeler et al. – Data Supplement MBH Neurons Regulate Cannabis-Induced Feeding*

**Supplemental Table S3. ANOVA analysis of locomotor activity data between home cage, open field, air, and cannabis rats.**

| ***Source*** | | ***F-Statistic*** | ***p-value*** | ***η_p_^2^*** |
| --- | --- | --- | --- | --- |
| *Distance ††* | |  |  |  |
|  | Drug (Air v. Cannabis) | 6.376 | **0.0243** | 0.313 |
|  | Location (Home Cage v. Open Field) | 53.03 | **<0.001** | 0.791 |
|  | Interaction | 6.266 | **0.0253** | 0.309 |
|  |  |  |  |  |
| *Time Spent Moving ††* | |  |  |  |
|  | Drug (Air v. Cannabis) | 6.612 | **0.0222** | 0.321 |
|  | Location (Home Cage v. Open Field) | 35.387 | **<0.001** | 0.717 |
|  | Interaction | 4.416 | 0.0542 | 0.240 |
|  |  |  |  |  |
| *Time Spent Still ††* | |  |  |  |
|  | Drug (Air v. Cannabis) | 5.978 | **0.0283** | 0.299 |
|  | Location (Home Cage v. Open Field) | 34.006 | **<0.001** | 0.708 |
|  | Interaction | 5.017 | **0.0418** | 0.264 |
|  |  |  |  |  |
| *Distance – Home Cage Group Only ††* | |  |  |  |
|  | Time (1hr-4hr) | 45.153 | **<0.001** | 0.849 |
|  | Drug (Air v. Cannabis) | 5.678 | **0.044** | 0.415 |
|  | Interaction | 0.510 | 0.679 | 0.060 |

*†† Two-Way ANOVA. n=12/group. Degrees of Freedom for each section: Drug(1), Location(1), Interaction(1), Model(3), Error(14). α = 0.05.*

*Wheeler et al. – Data Supplement MBH Neurons Regulate Cannabis-Induced Feeding*

**Supplemental Table S4. Fisher LSD means comparison of locomotor activity in open field and home cage, air and cannabis exposed rats.**

| ***Measurement*** | | | ***Mean Difference***  *(95% CI)* | ***q-value*** | ***p-value*** |
| --- | --- | --- | --- | --- | --- |
| *Distance* | | |  |  |  |
|  | Cannabis – HC | Cannabis – OF | -1431.6 (-2297.9 to -565.2) | -3.544 | **0.0032** |
|  | Air – OF | Cannabis – OF | -6.578 (-955.6 to 942.5) | -0.015 | 0.9884 |
|  |  | Cannabis – HC | 1425.0 (558.7 to 2291.4) | 3.528 | **0.0034** |
|  | Air – HC | Cannabis – OF | -2937.9 (-3886.9 to -1988.8) | -6.639 | **<0.0001** |
|  |  | Cannabis – HC | -1506.3 (-2372.7 to -640.0) | -3.729 | **0.0022** |
|  |  | Air – OF | -2931.3 (-3880.4 to -1982.3) | -6.625 | **<0.0001** |
|  |  |  |  |  |  |
| *Time Spent Moving* | | |  |  |  |
|  | Cannabis – HC | Cannabis – OF | 666.7 (427.2 to 906.2) | 5.970 | **<0.0001** |
|  | Air – OF | Cannabis – OF | -38.92 (-301.3 to 223.5) | -0.318 | 0.7551 |
|  |  | Cannabis – HC | -705.6 (-945.2 to -466.1) | -6.319 | **<0.0001** |
|  | Air – HC | Cannabis – OF | 279.7 (17.34 to 542.1) | 2.287 | **0.0383** |
|  |  | Cannabis – HC | -387.0 (-626.5 to -147.5) | -3.465 | **0.0038** |
|  |  | Air – OF | 318.6 (56.26 to 581.0) | 2.605 | **0.0208** |
|  |  |  |  |  |  |
| *Time Spent Still* | | |  |  |  |
|  | Cannabis – HC | Cannabis – OF | -666.8 (-905.7 to -427.9) | -5.986 | **<0.0001** |
|  | Air – OF | Cannabis – OF | 16.94 (-244.8 to 278.6) | 0.139 | 0.8916 |
|  |  | Cannabis – HC | 683.7 (444.8 to 922.6) | 6.138 | **<0.0001** |
|  | Air – HC | Cannabis – OF | -279.8 (-541.5 to -18.05) | -2.293 | **0.0379** |
|  |  | Cannabis – HC | 387.0 (148.1 to 625.9) | 3.474 | **0.0037** |
|  |  | Air – OF | -296.7 (-558.4 to -34.98) | -2.431 | **0.0291** |
|  |  |  |  |  |  |
| *Distance – Home Cage Group Only* | | |  |  |  |
|  | Air v. Cannabis | | 2877.8 (1689.1 to 4066.5) | 5.477 | **3.918E-4** |
|  |  | 1 hour | 2439.3 (282.97 to 4595.7) | 2.321 | **0.0281** |
|  |  | 2 hours | 2830 (673.6 to 4986.3) | 2.692 | **0.0120** |
|  |  | 3 hours | 2995.1 (838.8 to 5151.5) | 2.850 | **0.0083** |
|  |  | 4 hours | 3246.9 (1090.6 to 5403.2) | 3.090 | **0.0046** |

*Abbreviations: Home Cage (Sable Promethion Metabolic Chambers) – HC, Open Field – OC. Notes: Means compared using Fisher LSD; Home cage rats: n=6/experimental group, n=4/control group; Open field rats: n=4/group. α = 0.05.*

*Wheeler et al. – Data Supplement MBH Neurons Regulate Cannabis-Induced Feeding*

**Supplemental Table S5. ANOVA analyses for metabolic data of cannabis and air exposed rats.**

| ***Measurements*** | | | ***F-Statistic*** | ***p-Value*** | ***η_p_^2^*** |
| --- | --- | --- | --- | --- | --- |
| *Energy Expenditure (kcal/hr)* | | |  |  |  |
|  | Time (3min bins) | | 8.788 | **<0.001** | 0.239 |
|  | Drug (Air v. Cannabis) | | 5.535 | **0.026** | 0.165 |
|  | Interaction | | 1.171 | 0.314 | 0.040 |
|  |  | |  |  |  |
| *Respiratory Exchange Rate* | |  |  |  |  |
|  | Time (3min bins) | | 24.986 | **<0.001** | 0.472 |
|  | Drug (Air v. Cannabis) | | 0.817 | 0.374 | 0.028 |
|  | Interaction | | 1.724 | 0.084 | 0.058 |
|  |  | |  |  |  |
| *Rate of Oxygen Consumption (VO_2_)* | | |  |  |  |
|  | Time (3min bins) | | 9.381 | **<0.001** | 0.251 |
|  | Drug (Air v. Cannabis) | | 4.587 | **0.041** | 0.141 |
|  | Interaction | | 1.195 | 0.299 | 0.041 |
|  |  |  |  |  |  |
| *Rate of Carbon Dioxide Emission (VCO_2_)* | | |  |  |  |
|  | Time (3min bins) | | 6.920 | **<0.001** | 0.198 |
|  | Drug (Air v. Cannabis) | | 3.692 | 0.065 | 0.116 |
|  | Interaction | | 1.278 | 0.249 | 0.044 |

*Two-Way ANOVA repeated measures. n=6/experimental, n=4/control. Degrees of freedom Time(9), Interaction(9), Error Within-Subjects(252), Drug(1), Error Between-Subjects(28). α = 0.05.*

*Wheeler et al. – Data Supplement MBH Neurons Regulate Cannabis-Induced Feeding*

**Supplemental Table S6. ANOVA results for operant conditioning touches and touch latency of air and cannabis exposed rats**

|  |  | ***Number of Touches*** | | | ***Touch Latency*** | | |
| --- | --- | --- | --- | --- | --- | --- | --- |
| ***Measurement*** | | ***F-Statistic*** | ***p-value*** | ***η_p_^2^*** | ***F-Statistic*** | ***p-value*** | ***η_p_^2^*** |
| *Correct Touches* | |  |  |  |  |  |  |
|  | Time (1hr v. 2hr) | 2.828 | 0.124 | 0.220 | 0.016 | 0.903 | 0.002 |
|  | Drug (Air v. Cannabis) | 1.945 | 0.193 | 0.163 | 1.975 | 0.190 | 0.165 |
|  | Interaction | 6.455 | **0.029** | 0.392 | 0.495 | 0.498 | 0.047 |
|  |  |  |  |  |  |  |  |
| *Blank Touches* | |  |  |  |  |  |  |
|  | Time (1hr v. 2hr) | 14.82 | **0.003** | 0.597 | 0.355 | 0.564 | 0.034 |
|  | Drug (Air v. Cannabis) | 0.543 | 0.478 | 0.051 | 0.505 | 0.494 | 0.048 |
|  | Interaction | 10.66 | **0.008** | 0.516 | 0.890 | 0.368 | 0.082 |

Two-way ANOVA repeated measures, correct or blank touch and time. *η_p_^2^ = partial eta squared effect size. Degrees of Freedom: Time(1), Interaction(1), Within-Subjects Error(10), Drug(1), Between-Subjects Error(10). α = 0.05.*

*Wheeler et al. – Data Supplement MBH Neurons Regulate Cannabis-Induced Feeding*

**Supplemental Table S7. Tukey HSD means comparison of operant conditioning responses between air and cannabis exposed rats.**

| ***Measurement*** | | | | ***Mean Difference***  *(95% CI)* | ***q-value*** | ***p-value*** |
| --- | --- | --- | --- | --- | --- | --- |
| ***Number of Touches*** | | |  |  |  |  |
|  | *Correct Touches* | |  |  |  |  |
|  | | 1-hour Air | 1-hour Cannabis | -12.67 (-24.64 to -0.698) | 3.335 | **0.040** |
|  | |  | 2-hour Air | -2.0 (-9.338 to 5.338) | 0.859 | 0.557 |
|  | | 1-hour Cannabis | 2-hour Cannabis | 9.833 (2.495 to 17.17) | 4.222 | **0.014** |
|  | | 2-hour Air | 2-hour Cannabis | -0.833 (-12.80 to 11.14) | 0.219 | 0.880 |
|  | |  |  |  |  |  |
|  | *Blank Touches* | |  |  |  |  |
|  | | 1-hour Air | 1-hour Cannabis | -8.333 (-17.48 to 0.815) | 2.870 | 0.070 |
|  | |  | 2-hour Air | 1.00 (-4.388 to 6.388) | 0.585 | 0.688 |
|  | | 1-hour Cannabis | 2-hour Cannabis | 12.17 (6.779 to 17.55) | 7.116 | **5.13E-4** |
|  | | 2-hour Air | 2-hour Cannabis | 2.833 (-6.315 to 11.98) | 0.976 | 0.506 |
|  | |  |  |  |  |  |
| ***Touch Latency*** | | |  |  |  |  |
|  | *Correct Touch Latency* | |  |  |  |  |
|  | | 1-hour Air | 1-hour Cannabis | 28.73 (-12.02 to 69.49) | 2.222 | 0.147 |
|  | |  | 2-hour Air | 4.993 (-22.20 to 32.19) | 0.579 | 0.691 |
|  | | 1-hour Cannabis | 2-hour Cannabis | -7.153 (-34.35 to 20.04) | 0.829 | 0.571 |
|  | | 2-hour Air | 2-hour Cannabis | 16.59 (-24.16 to 57.34) | 1.283 | 0.386 |
|  | |  |  |  |  |  |
|  | | |  |  |  |  |
|  | *Blank Touch Latency* | |  |  |  |  |
|  | | 1-hour Air | 1-hour Cannabis | 12.15 (-10.98 to 35.28) | 1.655 | 0.269 |
|  | |  | 2-hour Air | 2.563 (-20.70 to 25.83) | 0.347 | 0.811 |
|  | | 1-hour Cannabis | 2-hour Cannabis | -11.36 (-34.63 to 11.90) | 1.539 | 0.302 |
|  | | 2-hour Air | 2-hour Cannabis | -1.780 (-24.91 to 21.35) | 0.243 | 0.867 |

*Two-way ANOVA repeated measures post-hoc means comparison. Degrees of freedom: 10. α = 0.05.*

*Wheeler et al. – Data Supplement MBH Neurons Regulate Cannabis-Induced Feeding*

**Supplemental Table S8. Tukey HSD means comparison of cannabis dose response in mice.**

| ***Measurement*** | | | ***Mean Difference***  *(95% CI)* | ***q-value*** | ***p-value*** |
| --- | --- | --- | --- | --- | --- |
| *Dose Response Mouse Model* | | |  |  |  |
|  | *Air* | 100mg | -0.122 (-0.470 to 0.226) | 1.471 | 0.730 |
|  |  | 200mg | -0.440 (-0.788 to -0.092) | 5.306 | **0.013** |
|  |  | 400mg | 0.312 (-0.036 to 0.660) | 3.762 | 0.085 |
|  |  |  |  |  |  |
|  | *100mg* | 200mg | -0.318 (-0.666 to 0.030) | 3.834 | 0.078 |
|  |  | 400mg | 0.434 (0.086 to 0.782) | 5.233 | **0.014** |
|  |  |  |  |  |  |
|  | *200mg* | 400mg | 0.752 (0.404 to 1.100) | 9.068 | **1.70E-4** |

*One-way repeated measures ANOVA, F_(3,12)_ = 14.065, ***p = 0.0003, η_p_^2^ = 0.779. n=5. α = 0.05.*

*Wheeler et al. – Data Supplement MBH Neurons Regulate Cannabis-Induced Feeding*

**Supplemental Table S9. ANOVA results for active cells during Ca^2+^ imaging of ARC neurons during feeding and anticipation, and air or cannabis exposure.**

| ***Measurement*** | | ***F-Statistic*** | ***p-Value*** | ***η_p_^2^*** |
| --- | --- | --- | --- | --- |
| *All Cells Per Condition* | |  |  |  |
|  | Drug (Air v. Cannabis) | 21.745 | **0.0186** | 0.879 |
|  | Feeding (Anticipation v. Consumption) | 0.4551 | 0.5483 | 0.132 |
|  | Interaction | 13.364 | **0.0354** | 0.817 |
|  |  |  |  |  |
| *Cells Active Only in Condition* | |  |  |  |
|  | Drug (Air v. Cannabis) | 21.745 | **0.0186** | 0.879 |
|  | Feeding (Anticipation v. Consumption) | 0.0224 | 0.8904 | 0.007 |
|  | Interaction | 13.364 | **0.0354** | 0.817 |

*Two-way ANOVA repeated measures. n=4. η_p_^2^ = partial eta squared effect size. Degrees of Freedom: Drug(1), Drug Error(3), Feeding Stage(1), Feeding Stage Error(3), Interaction(1), Interaction Error(3). α = 0.05.*

*Wheeler et al. – Data Supplement MBH Neurons Regulate Cannabis-Induced Feeding*

**Supplemental Table S10. Tukey HSD means comparison of active cell counts during air and cannabis exposure in Ca^2+^ imaging.**

|  |  | | ***Total Active Cells by Condition*** | | | ***Cells Active Only in Specified Condition*** | | |
| --- | --- | --- | --- | --- | --- | --- | --- | --- |
| ***Measurement*** | | | ***Mean Difference***  *(95% CI)* | ***q-Value*** | ***p-Value*** | ***Mean Difference***  *(95% CI)* | ***q-Value*** | ***p-Value*** |
| *Drug* | | |  |  |  |  |  |  |
|  | *Air v. Cannabis* | | -29.38 (-42.02 to -16.73) | 10.45 | **0.005** | -29.38 (-42.02 to -16.73) | 10.45 | **0.005** |
|  |  | |  |  |  |  |  |  |
| *Feeding* | | |  |  |  |  |  |  |
|  | *Anticipation v. Consumption* | | 0.375 (-12.27 to 13.02) | 0.134 | 0.931 | 0.375 (-12.27 to 13.02) | 0.134 | 0.931 |
|  |  | |  |  |  |  |  |  |
| *Drug*Feeding* | | |  |  |  |  |  |  |
|  | *Anticipation v. Consumption* | |  |  |  |  |  |  |
|  |  | Air | -11.5 (-43.61 to 20.61) | 1.612 | 0.337 | -4.0 (-21.89 to 13.89) | 1.007 | 0.528 |
|  |  | Cannabis | -2.75 (-34.86 to 29.36) | 0.386 | 0.803 | 4.75 (-13.14 to 22.64) | 1.195 | 0.460 |
|  | *Air v. Cannabis* | |  |  |  |  |  |  |
|  |  | Anticipation | -33.75 (-65.86 to -1.643) | 4.731 | **0.044** | -33.75 (-51.64 to -15.86) | 8.493 | **0.009** |
|  |  | Consumption | -25.0 (-57.11 to 7.107) | 3.505 | 0.089 | -25.0 (-42.89 to -7.114) | 6.291 | **0.021** |

*Two-way ANOVA repeated measures post-hoc Tukey HSD test, n=4. α = 0.05.*

*Wheeler et al. – Data Supplement MBH Neurons Regulate Cannabis-Induced Feeding*

**Supplemental Table S11. ANOVA results of AgRP DREADD infected mice given saline or CNO and cannabis or air.**

| ***Measurements*** | | | | ***[F-Statistic]***  ***t-Value*** | ***p-Value*** | ***η_p_^2^*** |
| --- | --- | --- | --- | --- | --- | --- |
| *30 minutes* | | | |  |  |  |
|  | Drug (Air v. Cannabis) | | | 5.406 | **0.037** | 0.294 |
|  | Injection (Saline v. CNO) | | | 1.948 | 0.186 | 0.130 |
|  | Interaction | | | 6.437 | **0.025** | 0.331 |
|  |  |  |  |  |  |  |
| *1 hour* | | | |  |  |  |
|  | Drug (Air v. Cannabis) | | | 6.614 | **0.023** | 0.337 |
|  | Injection (Saline v. CNO) | | | 4.257 | 0.060 | 0.247 |
|  | Interaction | | | 5.579 | **0.034** | 0.300 |
|  |  |  |  |  |  |  |
| *2 hours* | | | |  |  |  |
|  | Drug (Air v. Cannabis) | | | 8.374 | **0.013** | 0.392 |
|  | Injection (Saline v. CNO) | | | 1.800 | 0.203 | 0.122 |
|  | Interaction | | | 0.976 | 0.314 | 0.070 |

*Two-way ANOVA repeated measures, n=14. Degrees of freedom: Drug(1), Drug Error (13), Injection(1), Injection Error(13), Interaction(1), Interaction Error(13). α = 0.05.*

*Wheeler et al. – Data Supplement MBH Neurons Regulate Cannabis-Induced Feeding*

**Supplemental Table S12. Tukey HSD means comparison of AgRP DREADD infected mice given saline or CNO and cannabis or air.**

| ***Measurements*** | | | | | ***Mean Difference***  *(95% CI)* | ***q-Value*** | ***p-Value*** |
| --- | --- | --- | --- | --- | --- | --- | --- |
| *30 minutes* | | | | |  |  |  |
|  |  | Air – Saline | | Air – CNO | -0.725 (-2.541 to 1.091) | 1.220 | 0.404 |
|  |  |  | | Cannabis – Saline | -2.979 (-4.794 to -1.163) | 5.012 | **0.004** |
|  |  | Air – CNO | | Cannabis – CNO | 0.013 (-1.802 to 1.829) | 0.023 | 0.987 |
|  |  | Cannabis – Saline | | Cannabis – CNO | 2.267 (0.452 to 4.083) | 3.815 | **0.018** |
|  |  | |  |  |  |  |  |
| *1 hour* | | | | |  |  |  |
|  |  | Air – Saline | | Air – CNO | -0.768 (-3.136 to 1.599) | 0.992 | 0.495 |
|  |  |  | | Cannabis – Saline | -4.192 (-6.559 to -1.824) | 5.410 | **0.002** |
|  |  | Air – CNO | | Cannabis – CNO | -0.187 (-2.554 to 2.180) | 0.242 | 0.867 |
|  |  | Cannabis – Saline | | Cannabis – CNO | 3.236 (0.869 to 5.603) | 4.177 | **0.011** |
|  |  | |  |  |  |  |  |
| *2 hours* | | | | |  |  |  |
|  |  | Air – Saline | | Air – CNO | 0.430 (-4.024 to 4.885) | 0.295 | 0.838 |
|  |  |  | | Cannabis – Saline | -5.074 (-9.528 to -0.620) | 3.480 | **0.029** |
|  |  | Air – CNO | | Cannabis – CNO | -1.865 (-6.319 to 2.589) | 1.279 | 0.382 |
|  |  | Cannabis – Saline | | Cannabis – CNO | 3.639 (-0.815 to 8.093) | 2.496 | 0.101 |

*Two-way ANOVA repeated measures Tukey HSD post hoc results. α = 0.05.*
