# Supplementary material for: Cannabis Sativa targets mediobasal hypothalamic neurons to stimulate appetite
Source: Sci Rep. 2023 Dec 27;13:22970. doi: 10.1038/s41598-023-50112-5 (PMC10752887; doi:10.1038/s41598-023-50112-5)
Supplement: Supplementary file 2 — Supplementary Information. [file 41598_2023_50112_MOESM2_ESM.docx]

**Supplemental Information:**

Supplemental Methods:

*Diets:*

Rats and mice were maintained on standard rodent chow (3.41 kcal/g, LabDiet 5001) while in their home cages. When housed in the metabolic chambers, rats were maintained on a nutritionally equivalent diet (3.59 kcal/g, Research Diet D10012G) although the shape, color, and texture differed from the standard chow. A nutritionally complete HFD (HFD; 4.41 kcal/g, 1.71 kcal/g from fat, Research Diets Custom Formula) was used in the food zone in the calcium imaging experiments. All mice used in the calcium imaging experiments were exposed to 5 g of high fat diet placed in home cages prior to test days to reduce neophobia.

*Apparatus:*

Vapor chambers were constructed by attaching a cannabis vaporizer (Vaporizer 2.0, Vapir Inc., Santa Clara, CA.) to sealed positive ventilation rodent shoebox cages (Allentown Inc., Allentown, PA) as described previously [18]. The measurements of the Allentown positive ventilation rat shoebox cages were: base dimensions 14in x 10in x 7in; lid dimensions 14in x 10in x 2.5in; and the mouse shoebox cages were: base dimensions 11in x 7in x 5in; lid dimensions 11in x 7in x 2in. The flow rate of the VapirRise 2.0 was less than 1 L/min and cannabis plant matter was vaporized at 360 ^0^F.

*Calcium Imaging Data Analysis:*

Data were analyzed using methods described previously by Jennings *et. al* [40]. Briefly, a miniature microscope with an integrated LED (475 nm) was used to image GCaMP6s fluorescence in ARC neurons through an implanted microendoscope (8.4mm x 0.5mm). Images were acquired at 20 frames per second with the LED transmitting 0.1 to 0.2 mW of light on average using nVista HD Acquisition Software (version 2; Inscopix, Palo Alto, CA). Ca^2+^ imaging was synchronized with Ethovision behavioral data to track activity of ARC neurons when mice anticipated or consumed food.

All images were processed using the Mosaic software (version 1.0.0b; Inscopix, Palo Alto, CA). Each video sequence was spatially down sampled by a binning factor of 4. Video sequences were then corrected for movement using a single reference frame and the motion correction algorithm in Mosaic. Next, the mean z-projection image of the entire video as reference (F_0_) was created. This mean image was used to normalize the fluorescence signals to the average fluorescence of the whole frame. The video was temporally filtered by down sampling the video with a time binning factor of 4. Next, the Principle Component Analysis-Independent Component Analysis (PCA-ICA) algorithm was used on the ΔF/F_0_ videos, and single Ca^2+^ signals were isolated. Each signal identified was then manually inspected for each mouse and each condition. Finally, each individual unit was thresholded to remove background noise. Individual cells were then compared across conditions to determine activity state. Exported fluorescence values from this analysis were used to generate the heatmaps for each mouse and each condition. Specifically, the fluorescence values were calculated using the baseline (F_0_) values for the overall control condition. This was achieved by using the feeding control condition values as F_0_. The supplemental video file was extracted from the Mosaic software and then condensed temporally by a factor of 8 using Adobe Premier Pro 2020. FOV images were generated using the cell count function and images were hand traced to indicate cells activated following each condition.

*Slice Electrophysiology*:

To investigate the role of CB1R signaling on AgRP neurons, we performed uni- or bi-lateral pAAV.Syn.Flex.GCaMP6s.WPRE.SV40 (Addgene, #100845) injections on 6-8 week old *Agrp^tm1(cre)Low1^/J* mice (AP=-1.8, ML=0.3, DV=-5.7) for cell-type specific electrophysiological recordings. Following 2 weeks of viral incubation, mice were anesthetized with isoflurane, then underwent a pericardial perfusion with ice cold sucrose neuron protective solution for slicing (1°C) containing (in mM): 26 NaHCO_3_, 1.25 NaH_2_PO_4_, 2 KCl, 0.5 CaCl_2_, 5 MgCl_2_, 2 Na-pyruvate, 1 ascorbic acid, 10 D-glucose, and 220 sucrose 1 mM kynurenic acid. Animals were decapitated and brains were rapidly extracted into ice cold sucrose solution prior to preparation of brain slices in the same solution. Coronal slices (225 µm) in the range (Coronal plates 26-36; Bregma -2.12mm -4.52mm in: The rat brain in stereotaxic coordinates, Paxinos, George and Watson, Charles - 1986) were made using a Leica VT1200S vibratome in a slicing chamber filled with bubbling 1°C sucrose solution. Slices were then incubated in aCSF containing (in mM): 124 NaCl, 26 NaHCO_3_, 1 NaH_2_PO_4_, 2.5 KCl, 2.5 CaCl_2_, 2 MgCl_2_, and 10 D-glucose at near physiological temperature (32-34°C) for 1 hour, then in room temperature aCSF until electrophysiological recordings were conducted. Both the sucrose solution and the aCSF were bubbled with 95% O_2_/5% CO_2_ and contained 1 mM kynurenic acid (a glutamate receptor antagonist used to prevent potential excitotoxicity within the tissue).

Slices were secured with a nylon-strung, platinum harp in a submersion chamber mounted on an Olympus BX51WI microscope and visualized with a 60X (0.90 N.A.) water-immersion objective. Slices were perfused at a rate of 5-7 ml/min with artificial cerebrospinal fluid (aCSF), maintained at a temperature between 32-34° C, and bubbled with a 95% O_2_/5% CO_2_ gas. The AgRP cells were visually identified, using the appropriate fluorescence excitation and emission for the GFP fluorophore, and voltage-clamped (V_h_=-60mV with patch electrodes made from borosilicate glass capillary pipettes, containing (in mM) 130 CsCl, 4 NaCl, 0.5 CaCl_2_, 10 HEPES, 5 EGTA, 4 MgATP, 0.5 Na_2_GTP, and 5 QX-314. Internal solution was pH-adjusted to 7.2-7.3 with CsOH. For all recordings, perfusion aCSF was supplemented with 2mM kynurenic acid (general glutamate receptor blocker) to isolate sIPSCs in the recording. Electrode resistance was 4-10 MΩ for the AgRP neurons. Cells were excluded if access resistance changed by >20% across the duration of an experiment. All recordings were filtered at 10 kHz and acquired at 20kHz. Drugs were dissolved in aCSF+ kynurenic acid, bubbled with 95% O_2_/5% CO_2_ gas before being administered. Recordings were maintained for 5 minutes in each experimental condition, with the baseline condition being conducted for at least 5 minutes, or until a stable baseline was reached.

Spontaneous postsynaptic currents (sIPSCs) from the final two minutes of recording in each experimental condition were analyzed using Clampfit (Molecular Probes), using the function threshold search of event detection, with an amplitude threshold of 3 times the peak-to-peak amplitude of the noise, and then events were individually inspected with a further inclusion criterion of having a rise time at least 3 times faster than the decay time. Average frequency was determined including temporally overlapping events, then all non-overlapping events were averaged to calculate mean amplitude.

Kynurenic acid (1mM; Abcam; ab120064) was added to sucrose solution during brain extraction, and to aCSF during storage and recording. The CB1 agonist (+)-WIN 55,212-2 was purchased from Cayman Chemical (Cat. No. 10009023).

*Histology for verification of DREADD modified AgRP neurons in Agrp^tm1(cre)Low1^/J* *mice:*

To verify arcuate DREADD injection, *Agrp^tm1(cre)Low1^/J* mice were anesthetized under isoflurane and were perfused intracardially with PBS followed by 4% paraformaldehyde. The brains were removed and post-fixed overnight in the same fixative. The brains were cryoprotected by immersion in 30% sucrose in PBS at 4°C. 30-µm-thick coronal sections were cut by freezing microtome, mounted onto silane-coated glass slides, and cover slipped with Mounting Shield with DAPI (Vector Laboratories, Burlingame, CA, USA). Images were taken with Leica Microscope using MM AF software [20].

**Video S1.**

**Video S1: Representative Ca^2+^ Imaging Videos Depicting in vivo neuronal activity of MBH neurons in control or cannabis-treated mice.** Top left video: air anticipation; Top right video: air consumption; Bottom left video: cannabis anticipation; Bottom right video: cannabis consumption.
